# Multiple embryonic origins of nitric oxide synthase-expressing GABAergic neurons of the neocortex

**DOI:** 10.3389/fncir.2012.00065

**Published:** 2012-09-24

**Authors:** Lorenza Magno, Marcio G. Oliveira, Mariusz Mucha, Anna N. Rubin, Nicoletta Kessaris

**Affiliations:** Wolfson Institute for Biomedical Research and Department of Cell and Developmental Biology, University College LondonLondon, UK

**Keywords:** nNOS, interneurons, development, mouse, birthdating

## Abstract

Cortical GABAergic interneurons in rodents originate in three subcortical regions: the medial ganglionic eminence (MGE), the lateral/caudal ganglionic eminence (LGE/CGE), and the preoptic area (POA). Each of these neuroepithelial precursor domains contributes different interneuron subtypes to the cortex. Neuronal NOS (nNOS)-expressing neurons represent a heterogenous population of cortical interneurons. We examined the development of these cells in the mouse embryonic cortex and their abundance and distribution in adult animals. Using genetic lineage tracing in transgenic mice we find that nNOS type I cells originate only in the MGE whereas type II cells have a triple origin in the MGE, LGE/CGE, and POA. The two populations are born at different times during development, occupy different layers in the adult cortex and have distinct neurochemical profiles. nNOS neurons are more numerous in the adult cortex than previously reported and constitute a significant proportion of the cortical interneuron population. Our data suggest that the heterogeneity of nNOS neurons in the cortex can be attributed to their multiple embryonic origins which likely impose distinct genetic specification programs.

## Introduction

The gaseous biological messenger nitric oxide (NO) was originally described as a vasodilator (Furchgott and Zawadzki, 1980; Palmer et al., 1987) and has since been implicated in a variety of physiological processes. In the nervous system NO is involved in the regulation of cerebral blood flow, neurotransmission, synaptic plasticity and memory formation, modulation of neuroendocrine functions, and behavioral activity (Szabo, 1996). A role for NO in neurogenesis has also been proposed (Gibbs, 2003). NO is synthesized by the enzyme NO synthase (NOS) from the amino acid L-arginine. Three NOS-encoding genes have been identified and named according to the tissue in which they were first found: endothelial NOS (eNOS), neuronal NOS (nNOS), and the inducible form of NOS found in a variety of tissues (iNOS) (Alderton et al., 2001).

Cortical nNOS neurons are mainly GABAergic. They have been identified by immunohistochemical detection of nNOS and/or nicotinamide adenine dinucleotide phosphate diaphorase (NADPHd) staining (Dawson et al., 1991; Hope et al., 1991; Vincent, 2010). nNOS cortical neurons have been subdivided into two types according to the intensity of NOS/NADPHd staining: heavily labeled type I neurons that have large somata, and weakly labeled type II cells that have smaller somata (Hashikawa et al., 1994; Yan et al., 1996; Smiley et al., 2000; Lee and Jeon, 2005). Type I cells comprise around 0.5–2% of the cortical interneuron population (Kubota et al., 1994; Gonchar and Burkhalter, 1997). Type II cells are more numerous than type I in all species examined although their numbers vary in different cortical areas and across species (Yan et al., 1996; Smiley et al., 2000; Lee and Jeon, 2005). The two types of nNOS neurons have distinct but overlapping distributions within the cortex (Hashikawa et al., 1994; Kubota et al., 1994; Yan et al., 1996; Gonchar and Burkhalter, 1997; Smiley et al., 2000; Gotti et al., 2005; Lee and Jeon, 2005).

Detecting weakly-stained NOS cells has been challenging and consequently many studies have focussed on type I cells. The aspiny/sparsely spiny type I cells have round or oval cell bodies with bitufted, multipolar, or stellate morphologies (Valtschanoff et al., 1993; Gonchar and Burkhalter, 1997; Smiley et al., 2000; Gotti et al., 2005; Lee and Jeon, 2005). Type II cells have round cell bodies and at least some may correspond to neurogliaform cells (Smiley et al., 2000; Price et al., 2005; Karagiannis et al., 2009). The two populations have distinct neurochemical content and physiological features and are therefore thought to represent two functionally different neuronal populations within the cortical network (Dawson et al., 1991; Kubota et al., 1994, 2011; Gonchar and Burkhalter, 1997; Smiley et al., 2000; Lee and Jeon, 2005; Karagiannis et al., 2009).

Some characteristics of cortical interneurons are specified at the time when these cells are born. A number of studies have examined where interneurons are generated in order to understand how heterogeneity is established (Wonders and Anderson, 2006; Gelman and Marin, 2010; Rubin et al., 2010; Gelman et al., 2011). Unlike cortical pyramidal neurons which are born sequentially from a common pool of local precursors, interneurons are born outside the cortex, and migrate into the cortex during embryogenesis (Wonders and Anderson, 2006). Genetic fate-mapping has confirmed that the two main sources of cortical interneurons are the medial ganglionic eminence (MGE) and the lateral/caudal ganglionic eminence (LGE/CGE) in the subpallium (Fogarty et al., 2007; Miyoshi et al., 2007, 2010; Xu et al., 2008; Sousa et al., 2009; Rubin et al., 2010). The preoptic area (POA) has also been shown to generate small numbers of interneurons for the cortex (Gelman et al., 2009, 2011). Cortical interneurons originating from these three spatially segregated precursor pools are born at different times and have distinct neurochemical phenotypes and physiological properties. The origin of cortical nNOS cells has not been determined.

In this study we examined the timing of generation of nNOS-expressing cortical interneurons and emergence of the two subtypes. Using a series of transgenic mice that genetically label distinct neuroepithelial domains in the subpallium we identified the embryonic origin of the two populations and characterized their distribution patterns and neurochemical profiles within the adult somatosensory cortex.

## Materials and methods

### Transgenic mice

All transgenic mouse lines used in this study have been described previously: *Nkx2.1*–*Cre*^*Tg*^, *Lhx6-Cre*^*Tg*^, *Nkx6.2-Cre*^*Tg*^ (Kessaris et al., 2006; Fogarty et al., 2007), *Dlx1-lox-Venus-lox*^*Tg*^ (Rubin et al., 2010), *Nkx5.1-Cre*^*Tg*^ (Gelman et al., 2009), *Shh-GFPCre*^*KI*^ (Harfe et al., 2004). Herein we refer to them as *Nkx2.1-Cre*, *Lhx6-Cre*, *Nkx6.2-Cre*, *Dlx1-Venus*^*fl*^, *Nkx5.1-Cre, and Shh-Cre*, respectively. Mice were maintained on a mixed C57BL/6/CBA background at the Wolfson Institute for Biomedical Research, University College London, and the National Institute for Medical Research, London, in accordance with United Kingdom legislation.

Three reporter mice for Cre recombinase have been used in this study: *Rosa26R–Green Fluorescent Protein (GFP) (R26R-GFP)* (Mao et al., 2001), *Rosa26R–yellow fluorescent protein (YFP) (R26R-YFP)* (Srinivas et al., 2001), and *Rosa26R–LacZ* (Soriano, 1999). Upon Cre-mediated recombination, the three mice express GFP, YFP, and β-galactosidase, respectively, under control of the Rosa26 promoter.

### *In situ* hybridization

Tissue preparation and *in situ* hybridization were carried out as previously described (Rubin et al., 2010). To detect *nNos* transcripts we used several different RNA probes that recognize the full length *nNos*α transcript and one or more of its splice variants (*nNos*β, *nNos*γ, *nNos*μ, and *nNos*-2). All probes gave comparable results (data not shown). We present images using a probe spanning exon 2 of the mouse *nNOS* gene which encodes the PDZ domain (PSD-95 discs large/ZO-1 homology domain), a unique feature of *nNos* that distinguishes it from *eNos* and *iNos*. This probe spans 660 bp from the mouse *nNos* gene and detects *nNos*α, *nNos*μ, and *nNos*-2 (Alderton et al., 2001). It was generated by PCR amplification from genomic DNA using the following primers: 5′-CCAACGTCATTTCTGTCCGTC-3′ and 5′-TTCCTGTGTCTTTCATCTCTGC-3′. The PCR product was cloned into pCRII-TOPO (Invitrogen). The plasmid was linearized with *SpeI* and an antisense digoxigenin (DIG)-labeled RNA probe was transcribed using T7 RNA polymerase (Promega).

### Immunohistochemistry

Unless otherwise stated, immunohistochemical detection of calbindin (CB), calretinin (CR), parvalbumin (PV), somatostatin (SST), neuropeptide Y (NPY), reelin (RLN), nNOS, GFP/YFP, and β-galactosidase (β-gal) was carried out as described previously (Rubin et al., 2010). To amplify the nNOS signal and detect the weak-expressing type II cells we used the Vectastain ABC kit (Vector Laboratories) followed by either Tyramide-Cy3 (Perkin Elmer) as a fluorescent enzyme substrate or DAB reagent (Vector Laboratories) as a chromogenic substrate, according to manufacturers' instructions. Briefly, endogenous peroxidase activity was quenched with 0.6% H_2_O_2_ for 20 min and anti-nNOS was applied overnight. A biotin-conjugated secondary antibody was used to detect the primary anti-nNOS antibody followed by the Avidin/Biotinylated enzyme Complex (ABC) (prepared according to manufacturer's instructions). Tyramide-Cy3 (Perkin Elmer) (1:300 in amplification buffer) or DAB substrate reagent (Vector Laboratories) were applied for 3 min or 1 min, respectively, before sections were mounted.

Primary antibodies used were the following: rat anti-GFP IgG2a (1:1000; Nacalai Tesque; Cat no. 0440484); rabbit anti-β-galactosidase (1:2000; MP Biomedicals; Cat no. 55976); mouse anti-CB (1:1000; Swant; Cat no. 300); rabbit anti-CR (1:1000; Swant; Cat no. 7699/3H); mouse anti-PV (1:1000; Chemicon/Millipore; Cat no. MAB1572); rabbit anti-SST (1:200 Peninsula Laboratories; Cat no. T410300); rabbit anti-NPY (1:1000, ImmunoStar; Cat no. 22940); mouse anti-RLN (1:200) (kindly provided by A. Goffinet). To detect nNOS we used several different antibodies that recognize different regions of the nNOS protein in an effort to identify the optimal conditions for detecting nNOS type II cells. These included the following: rabbit anti-nNOS that recognizes 195 amino acids from N-terminus of the rat nNOS (1:500; Invitrogen; Cat no. 61-7000), sheep anti-nNOS generated against recombinant rat nNOS [1:1000; (kindly provided by P. Emson) (Herbison et al., 1996)], mouse monoclonal anti-nNOS that recognizes amino acids 1095–1289 from the C terminus of human nNOS (1:200; BD Biosciences; Cat no. N31020-050), and rabbit anti-nNOS generated against amino acids 1419–1433 from the C terminus of human nNOS (1:1000; Immunostar; Cat no. 24287). All antibodies gave comparable results. Data presented in this study were generated using the rabbit anti-nNOS (Immunostar) and the sheep anti-nNOS (Herbison et al., 1996).

Secondary antibodies used were biotin-conjugated donkey anti-rabbit IgG (1:500; Millipore), biotin-conjugated donkey anti-sheep IgG (1:200; Thermo Scientific), AlexaFluor 488- and AlexaFluor 568-conjugated goat anti-rabbit IgG, or goat anti-rat IgG or goat anti-mouse IgG (all used at 1:750; Invitrogen).

### EdU birthdating

5-ethynyl-2′-deoxyuridine (EdU, Molecular Probes) was dissolved in sterile PBS at 2 mg/ml. Pregnant females were administered five intraperitoneal injections of EdU (10 mg/Kg body weight) at 2 h intervals starting at 10:00 am. The pups were perfused at P30 with 4% PFA and tissue was further fixed for 45 min at room temperature by immersion in the same solution. EdU detection was carried out after nNOS immunohistochemistry using the Click-iT EdU Alexa Fluor 647 Imaging Kit (Molecular Probes) according to manufacturer's instructions. Briefly, following detection of nNOS, the sections were incubated in Click-iT EdU reaction cocktail (prepared according to manufacturer's instructions) in the dark for 45 min before being washed and mounted.

### Quantification

The extent of co-localization between nNOS and other markers was determined as previously described (Fogarty et al., 2007). In all experiments quantification was carried out in the somatosensory cortex between Bregma position 0.74 and −1.22 mm. Cells were counted in a defined area spanning the pial–white matter extent of the cortex (450 μm width and 30 μm depth). In some cases this was subdivided into 10 equal bins along the dorso-ventral axis and the number of cells in each bin was determined. For all quantification experiments a minimum of three mice were used. Counts were performed on at least three consecutive sections (six hemispheres) from each mouse. Results are expressed as mean ± standard error of the mean (SEM). Graphical representations of the data and statistical analysis were performed using GraphPadPrism for Microsoft Windows.

## Results

### nNOS-expressing interneurons in the developing cortex

**Figure 1:**
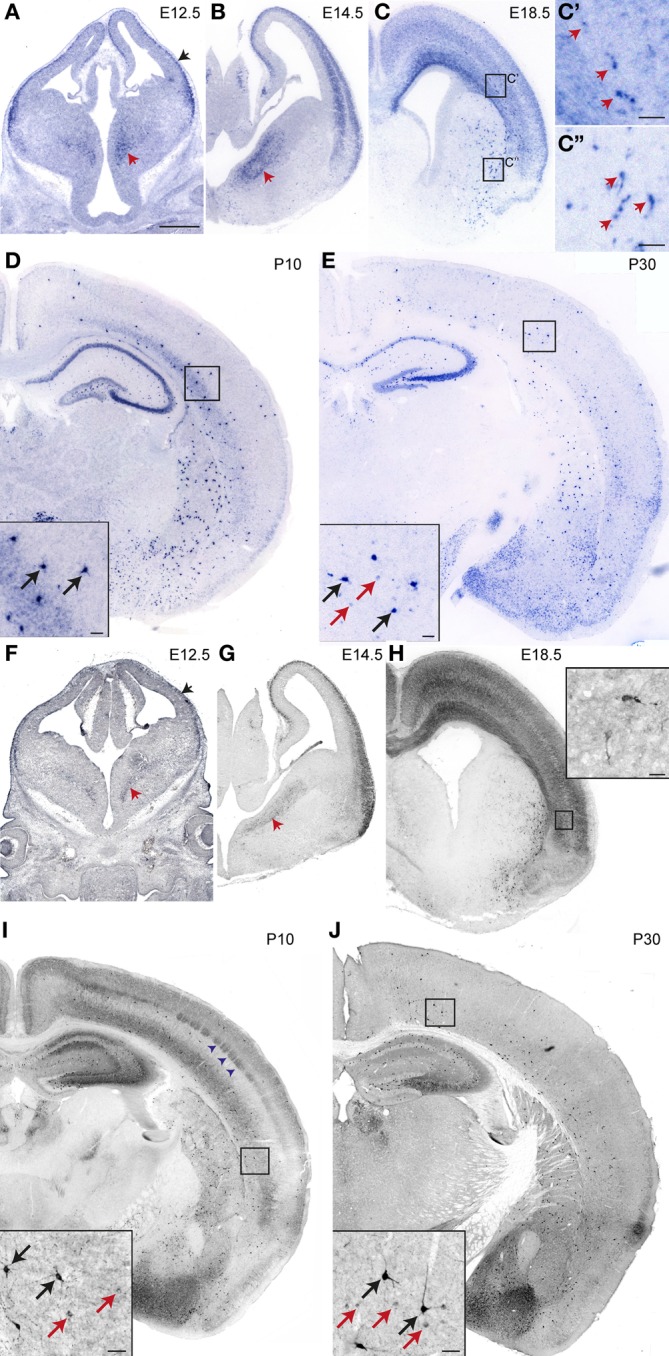
**Developmental expression of nNOS in the neocortex. (A–E)** *nNos* mRNA expression at different embryonic and postnatal stages. Black and red arrowheads in **(A)** and **(B)** point to expression in the superficial cortex and the ventral MGE, respectively. Arrowheads in **(C')** and **(C”)** point to cells expressing high levels of nNOS in the cortex and striatum, respectively. **(F–J)** nNOS protein expression at different embryonic and postnatal stages. Type I and type II cells are marked by black and red arrows, respectively in **(I)** and **(J)**. Scale bars: **A–J**, 500 μm. **C, C”** and inserts in **D**, **E**, **I**, **J**, 50 μm. Insert in **H**, 20 μm.

We examined *nNos* mRNA and protein expression in the telencephalon at embryonic and postnatal stages. We detected nNOS transcripts in the cortex at E12.5 (Figure 1A). At this stage, expression was confined to the marginal zone and/or the cortical plate (black arrowhead in Figure 1A). This may correspond to Cajal-Retzius and cortical plate cells as previously described in the rat (Bredt and Snyder, 1994; Santacana et al., 1998). *nNos* was also expressed in scattered cells in the subventricular zone of the MGE (red arrowheads in Figures 1A,B) which is one of the sources of interneurons for the cortex. A clear but transient expression in the cortical plate was observed at E14.5, E18.5 (Figures 1B,C), and P5 (not shown). This was largely downregulated by P10 (Figure 1D). Presumptive cortical interneurons intensely labeled for *nNos* appeared scattered within the subventricular/intermediate zone of the lateral cortex at E18.5 (Figures 1C,C'). Later on these cells populated the entire medio-lateral extent of the cortex and the hippocampus and resided mainly within the deeper layers in postnatal animals (Figures 1D,E). Cells expressing high levels of *nNos* were also observed in the striatum (Figures 1C–E). A similar expression pattern was observed by nNOS immunohistochemistry (Figures 1F–J). Strong immunolabeling of nNOS protein was detected in a few scattered cells in the cortex at E18.5 and these increased in numbers thereafter (black arrows in Figures 1I,J). These are thought to represent nNOS type I cortical interneurons which express high levels of nNOS (Hashikawa et al., 1994; Yan et al., 1996; Smiley et al., 2000; Lee and Jeon, 2005). We could detect weakly-labeled putative nNOS type II cells from P10 onwards (red arrows in Figures 1I,J). We could not determine whether this represented the true onset of nNOS expression or the timing of appearance of type II cells because at earlier stages the strong nNOS signal in the cortical plate may have masked any weak expression in interneurons. A transient diffuse nNOS signal was observed in the deep cortical layers and in layer IV at P10 (Figure 1I). The barrel-like nNOS immunolabeling in layer IV (blue arrowheads in Figure 1I) may correspond to staining in the barrel centers, which are formed by afferents from the thalamus, or the barrel walls, which contain layer IV neurons. We could not detect *nNOS* mRNA expression in layer IV at this stage (Figure 1D) suggesting that afferent inputs from the thalamus, where nNOS immunoreactivity has been observed (Terada et al., 2001), may account for the signal.

**Figure 2:**
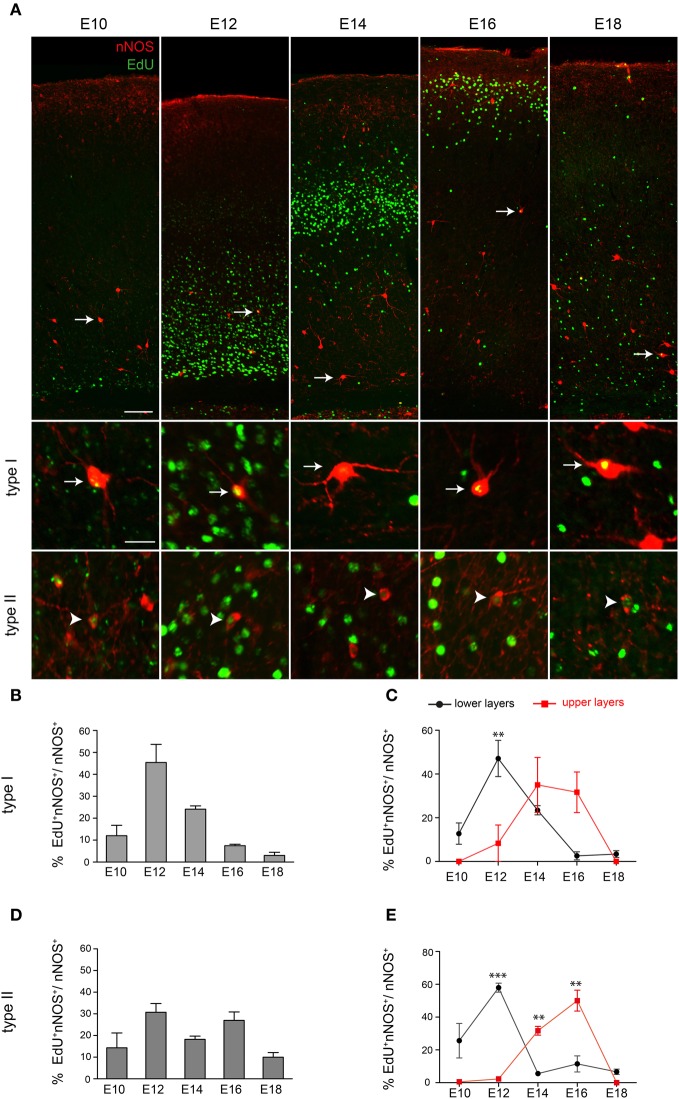
**Neurogenesis period of cortical nNOS cells. (A)** nNOS-positive neurons and EdU-incorporating cells at P30 following EdU administration at different developmental stages. White arrows and arrowheads indicate nNOS-immunoreactive neurons colabeled with EdU. These are shown at higher magnification in the lower panel for type I (arrows) and type II cells (arrowheads). **(B)** Histogram showing the EdU labeling index for nNOS type I cells. **(C)** Neurogenesis periods of nNOS type I cells residing in lower and upper cortical layers. A significant difference is detected at E12. ^**^P < 0.01 (Two-way ANOVA with Bonferroni *post-hoc* test). **(D)** Histogram showing the EdU labeling index for nNOS type II cells. **(E)** Neurogenesis periods of nNOS type II cells residing in lower and upper cortical layers. A significant difference is detected at E12, E14, and E16. ^**^P < 0.01 ^***^P < 0.001 (Two-Way ANOVA with Bonferroni *post-hoc* test). Error bars indicate SEM. Scale bars: **A**, upper panel, 100 μm, lower panel, 20 μm.

Our data show that interneurons expressing nNOS appear in the cortex just before birth. This suggests that either nNOS cortical interneurons are born late and enter the cortex late or that nNOS activation occurs in these cells only after they invade the cortex. To distinguish between the two possibilities we birthdated nNOS interneurons using EdU labeling at different embryonic stages and analysis at P30. nNOS type I and type II cells incorporating EdU could be detected at all stages examined (E10, E12, E14, E16, E18) (Figure 2A). Quantification of the extent of co-localization between EdU and nNOS showed that the majority of nNOS type I cells are born between E12 and E14 with E12 being the peak generation time (Figure 2B). Neurogenesis of type II cells spanned a longer period of time (Figure 2D). We also determined whether laminar fate is dependent on birthdate. We subdivided the cortex into two equal halves along the dorso-ventral axis and quantified the extent of EdU/nNOS colocalization. Although upper and lower layer nNOS neurons had overlapping neurogenesis periods, they had different peak generation times with most lower layer nNOS neurons being born earlier than the bulk of upper layer ones (Figures 2C,E). Collectively, our data indicate that nNOS cortical interneurons are born early during embryogenesis but express their definitive marker nNOS at later stages. Type II cells have a more protracted neurogenesis period compared to type I cells. In addition, settling of nNOS interneurons within the cortex occurs in an inside-out manner, as previously described for other cortical interneuron subtypes (Fairen et al., 1986; Rymar and Sadikot, 2007).

### nNOS type I and type II interneuron distribution in the adult somatosensory cortex

**Figure 3:**
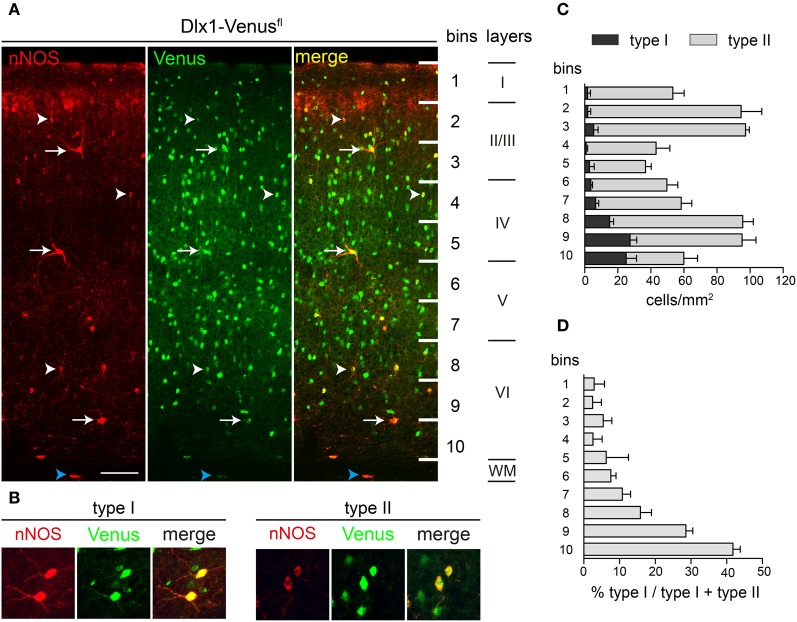
**nNOS-expressing type I and type II cells in the adult somatosensory cortex. (A)** nNOS and Venus expression in the somatosensory cortex of *Dlx1-Venus*^*fl*^ mice. Arrows and arrowheads point to type I and type II cells respectively. Blue arrowheads mark nNOS-expressing cells in the white matter (WM). **(B)** High magnification images of type I and type II cells. Note the strong labeling for nNOS in the soma and the processes of type I cells and the pale nNOS staining in type II cells. **(C)** Quantification of type I and type II cell densities across cortical bins. **(D)** Abundance of type I cells across bins as a fraction of the total nNOS cells in each bin. Error bars indicate SEM. Scale bars: **A**, 50 μm; **B**, 20 μm.

We examined in detail the distribution of nNOS-expressing interneurons in the adult cortex using immunohistochemistry for nNOS and Venus in *Dlx1-Venus*^*fl*^ transgenic mice. These mice express Venus in all GABAergic interneurons of the cortex (Rubin et al., 2010) and therefore allow us to definitively distinguish GABAergic nNOS interneurons from the few nNOS-expressing pyramidal cells which are found in upper cortical layers (Figure 3A). All nNOS-expressing neurons in the cortex, with the exception of a few cells in layer II, coexpressed Venus in the *Dlx1-Venus*^*fl*^ transgenic mice confirming their GABAergic phenotype (Figure 3A). In addition to the gray matter, nNOS/Venus coexpressing cells were also found in the white matter of the cortex (blue arrowheads in Figure 3A). nNOS type I cells showed distinctive immunoreactivity for nNOS: the cell body was intensely labeled and processes were clearly visible (Figure 3B). Type II cells had weaker immunoreactivity, processes were often indistinguishable and the cell body had uneven staining (Figure 3B). To quantify the density and distribution of nNOS interneurons in the cortex we counted double labeled Venus/nNOS type I and type II cells in different cortical layers: for this we subdivided the cortex into 10 equal bins along the white matter-pial axis and counted the double positive cells within each bin. Most type I cells were located within layer VI whereas type II cells had a maximum density in layers II/III and VI (Figure 3C). nNOS type I cells were rare and represented ~2.5% of the total GABAergic interneuron population whereas type II cells represented ~17% of cortical interneurons. Type II cells were ~6.5 fold more abundant than type I cells in the somatosensory cortex although their relative abundance varied across different layers (Figure 3D). Our data indicate that there exists a clear heterogeneity of nNOS-expressing interneurons that is based not only on the level of expression of nNOS but also on the distribution and abundance of these cells within the cortex.

### Coexpression of nNOS with other interneuron markers in type I and type II cells

**Figure 4:**
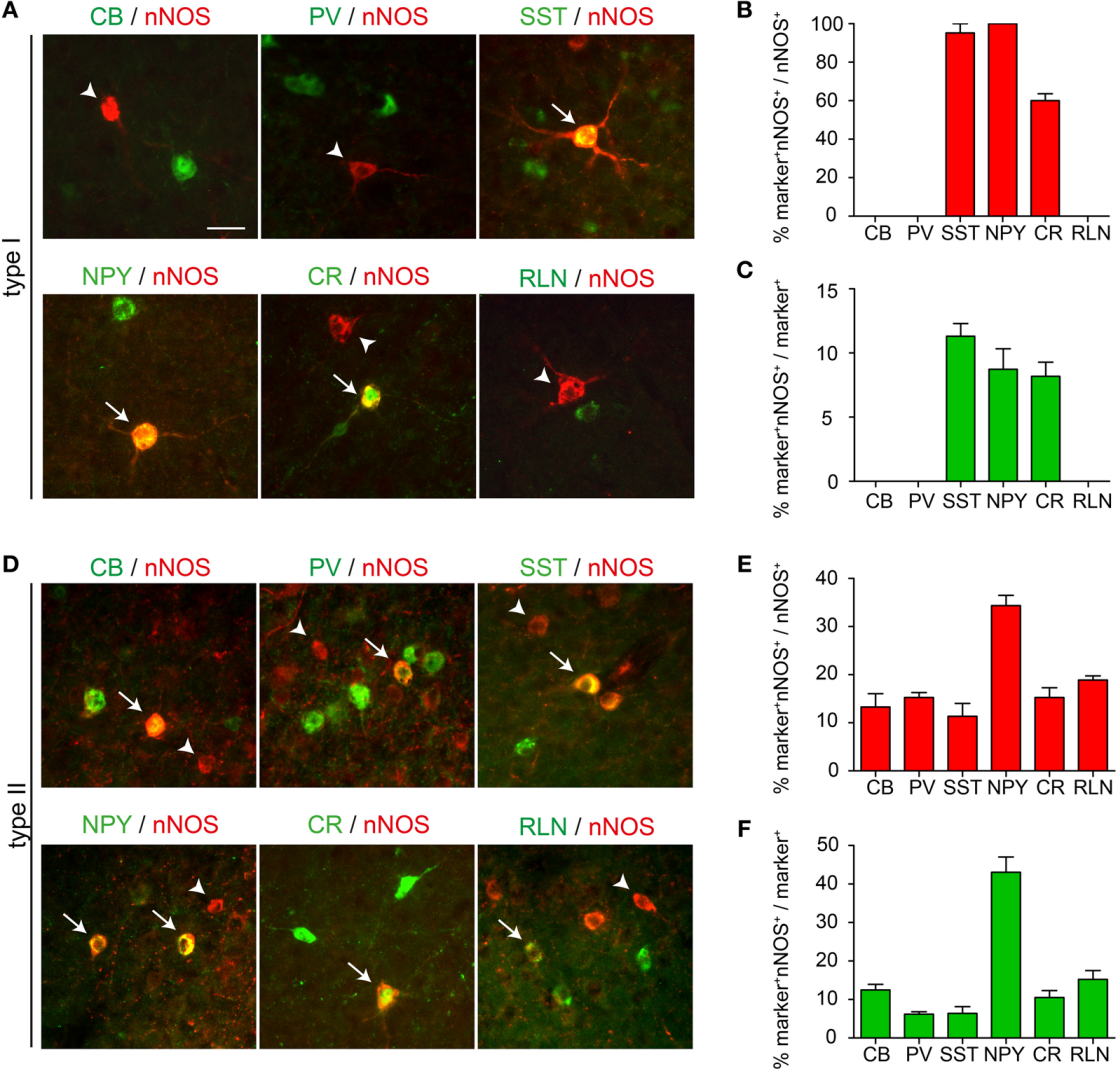
**Coexpression of nNOS with other interneuron markers. (A)** Expression of CB, PV, SST, NPY, CR, or RLN in nNOS type I cells. **(B)** Quantification of the extent of coexpression between nNOS and the different interneuron markers in type I cells. **(C)** The number of marker^+^ nNOS type I cells is presented as a percentage of the total number of marker^+^ cells. **(D)** Expression of CB, PV, SST, NPY, CR, or RLN in nNOS type II cells. **(E)** Quantification of the extent of coexpression between nNOS and the different interneuron markers in type II cells. **(F)** The number of marker^+^ nNOS type II cells is presented as a percentage of the total number of marker^+^ cells. Error bars indicate SEM. Scale bar: 20 μm.

To determine whether nNOS type I cells can be subdivided further based on expression of other neurochemical markers, we examined the extent of co-localization between nNOS and CB, PV, SST, NPY, CR, and RLN. To avoid potential bleed-through artifacts arising from the strong fluorescence of type I cells detected by our amplification method, we quantified the extent of marker coexpression in nNOS in type I cells using regular immunohistochemistry whereby the primary anti-nNOS antibody was detected by an Alexa-conjugated secondary antibody. In the absence of signal amplification, only type I cells can be detected in the cortex. We found no co-localization between nNOS and CB, PV, or RLN in type I cells (Figures 4A,B). In contrast, all nNOS type I cells coexpressed NPY, nearly all coexpressed SST and ~60% coexpressed CR (Figures 4A,B). Coexpression of nNOS with NPY and SST was also confirmed by *in situ* hybridization for NPY or SST followed by immunohistochemistry for nNOS (not shown). nNOS type I cells represented less than 10% of the total population of cortical NPY, SST, or CR interneurons (8.7 ± 1.6% for NPY, 11.3 ± 1% for SST, and 8.2 ± 1.1% for CR) (Figure 4C). In contrast to nNOS type I cells, type II cells showed some co-localization with all markers examined (Figures 4D–F). A large number of nNOS type II cells coexpressed NPY (Figure 4E).

### The embryonic origin of cortical nNOS interneurons

The origin of cortical interneurons has been identified and it is clear that distinct interneuron cohorts originate from different neuroepithelial domains in the developing telencephalon. To a large extent, the apparent heterogeneity of interneurons observed in the adult cortex is laid down early on when these cells are born. We therefore examined whether the two types of nNOS interneurons originate from different neuroepithelial regions. For this we made use of a series of transgenic mice that express Cre recombinase in different domains of the developing telencephalon. When crossed to suitable reporters, these mice indelibly label the entire cell lineage originating in each domain. The mice used for lineage tracing in this study were the following: *Lhx6-Cre*/*R26R-YFP* which label all MGE-derived interneurons (Fogarty et al., 2007), *Nkx2.1-Cre*/*R26R-GFP* and *Nkx6.2-Cre*/*R26R-GFP* which label different populations of MGE-derived interneurons (as well as POA-derived neurons) (Fogarty et al., 2007), *Dlx1-Venus*^*fl*^/*Nkx2.1-Cre* which label all LGE/CGE-derived cells (Rubin et al., 2010), and *Nkx5.1-Cre/R26R-YFP* and *Shh-Cre/R26R-LacZ* which label different subpopulations of POA-derived cortical interneurons (Gelman et al., 2009, 2011). Herein we refer to them as *Lhx6-Cre*/*YFP*, *Nkx2.1-Cre/GFP*, *Nkx6.2-Cre/GFP*, *Dlx1-V*^*fl*^/*Nkx2.1-Cre*, *Nkx5.1-Cre/YFP*, and *Shh-Cre/LacZ*. We examined the co-localization of nNOS with GFP/YFP/Venus/β-gal (referred to as XFP/β-gal) and quantified this for type I and type II cells in the somatosensory cortex in adult mice. Where necessary, immunodetection of type I cells without signal amplification for nNOS was used to avoid any bleed-through artifacts arising from the strong fluorescence of type I cells. This was carried out in *Nkx2.1-Cre*/*GFP*, *Nkx6.2-Cre*/*GFP*, *Nkx5.1-Cre/YFP*, and *Shh-Cre/LacZ* transgenic mice. The strong Venus/YFP fluorescence in *Dlx1-V*^*fl*^/*Nkx2.1-Cre* and *Lhx6-Cre*/*YFP* (two copies of the YFP reporter) transgenic mice circumvented all bleed-through problems.

**Figure 5:**
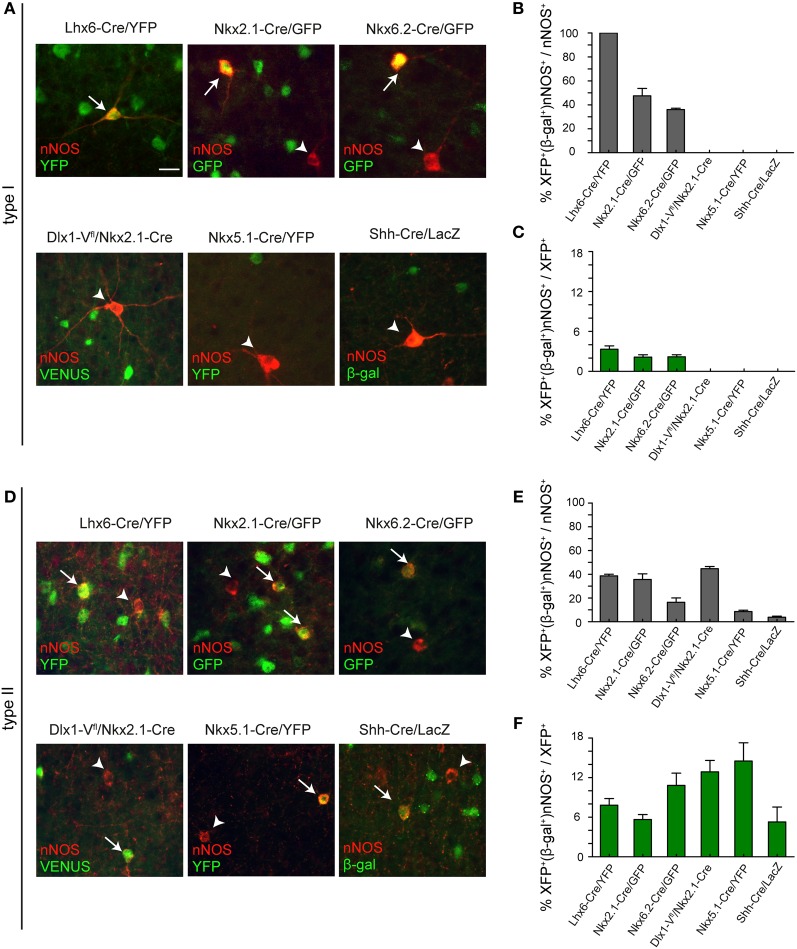
**Embryonic origin of type I and type II nNOS cortical interneurons. (A)** Coexpression of nNOS and GFP/YFP/Venus/β-gal in type I cells in different transgenic mouse lines. Arrows and arrowheads point to double and single-labeled type I cells, respectively. **(B)** Contribution of different progenitor pools to type I cortical interneurons. The extent of co-localization between nNOS and GFP/YFP/Venus/β-gal in type I cells is shown as a percentage of the total nNOS type I cells **(B)** or as a percentage of the total GFP/YFP/Venus/ β-gal-expressing interneurons **(C)**. **(D)** Coexpression of nNOS and GFP/YFP/Venus/β-gal in type II cells in different transgenic mouse lines. Arrows and arrowheads point to double and single-labeled type II cells, respectively. **(E)** Contribution of different progenitor pools to type II cells. The extent of co-localization between nNOS and GFP/YFP/Venus/β-gal in type II cells is shown as a percentage of the total nNOS type II cells (**E**) or as a percentage of the total GFP/YFP/Venus/β-gal-expressing interneurons **(F)**. Error bars indicate SEM. Scale bar: 20 μm.

All type I cells coexpressed YFP in *Lhx6-Cre/YFP* mice indicating that they are all derived from the MGE (Figures 5A,B). In previous studies we were able to distinguish between the contribution of the dorsal MGE (dMGE) and the rest of the MGE neuroepithelium using transgenic mice expressing *Nkx6.2-Cre* and *Nkx2.1-Cre* (Fogarty et al., 2007). *Nkx6.2-Cre* mice activate the *R26R-GFP* reporter mainly in the dMGE with only scattered activation in the rest of the MGE. In contrast, *Nkx2.1-Cre* transgenic mice label most of the MGE with the exception of a small dorsal domain that expresses high levels of *Nkx6.2*. Both mice express Cre in the POA. The two transgenic mice therefore have complementary albeit partly overlapping patterns of Cre expression. We found that, unlike *Lhx6-Cre/YFP* transgenic mice where all nNOS type I cells coexpressed YFP, in *Nkx2.1-Cre/GFP* or *Nkx6.2-Cre/YFP* transgenic mice less than 50% of the type I cells co-localized with GFP/YFP (Figures 5A,B). This suggests that there is contribution of nNOS type I cells from both the *Nkx6.2-Cre*-expressing dMGE neuroepithelium as well as the rest of the MGE. There was no Venus or YFP expression in nNOS type I cells in the LGE/CGE- or the POA-tracing mice confirming that these two regions do not generate cortical nNOS type I cells (Figures 5A,B). Consistent with nNOS type I cells being very few, they represented less than 5% of the cortical interneurons generated from the MGE which is the main source of interneurons for the cortex (Figure 5C).

**Figure 6:**
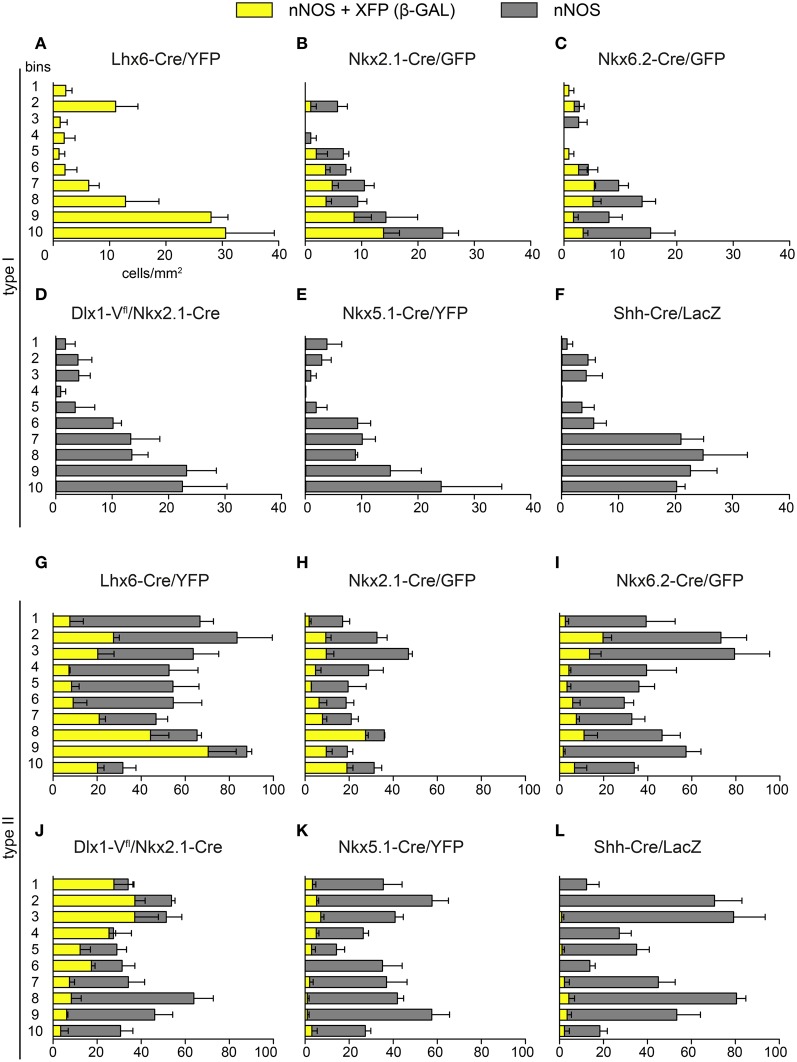
**Laminar distribution of nNOS immunoreactive cells originating in different embryonic neuroepithelial regions.** The density of type I **(A–F)** and type II cells **(G–L)** coexpressing GFP/YFP/Venus/β-gal (yellow) as well as the total number of nNOS-expressing cells (gray) across cortical bins are shown for each transgenic mouse line. Error bars indicate SEM.

In contrast to type I cells, type II cells appear to have a triple MGE, LGE/CGE, and POA origin (Figures 5D–F). Most of these cells are generated in the MGE and the LGE/CGE. The POA contributes only a small number (Figures 5D–F). Intriguingly, the contribution of *Nkx2.1-Cre*-expressing precursors to the total population of nNOS type II cells is similar to that of the entire MGE in *Lhx6-Cre/YFP* mice (Figure 6E). This suggests that most MGE-derived type II cells are born outside the dMGE neuroepithelium.

### Laminar distribution of nNOS interneurons from different embryonic origins

Previous genetic fate-mapping work had shown that interneurons that have different embryonic origins settle in different layers within the adult cortex (Miyoshi et al., 2007, 2010; Gelman et al., 2009, 2011; Rubin et al., 2010). We examined the laminar distribution of nNOS/XFP/β-gal cells in the six transgenic mouse lines described above (Figures 6A–L). Type I cells originating from *Lhx6*-expressing precursors and representing the entire type I population settle within the lower layers of the cortex (Figure 6A). Whilst *Nkx2.1*-derived type I cells had a tendency to populate middle and lower layers, *Nkx6.2*-derived type I cells populated mainly middle layers (Figures 6B,C). MGE- and LGE/CGE-derived type II cells occupied respectively lower and upper layers of the cortex (Figures 6G,J), as previously described for other cortical interneuron subtypes originating from these two regions (Xu et al., 2004; Miyoshi et al., 2007, 2010; Rubin et al., 2010). A tendency for *Nkx2.1*-derived type II cells to populate lower layers than *Nkx6.2*-derived ones was also observed (Figures 6H,I). The few nNOS type II cells originating from *Nkx5.1*- and *Shh*-expressing domains occupied different cortical layers, suggesting that they represent distinct populations of type II neurons (Figures 6K,L).

## Discussion

**Figure 7:**
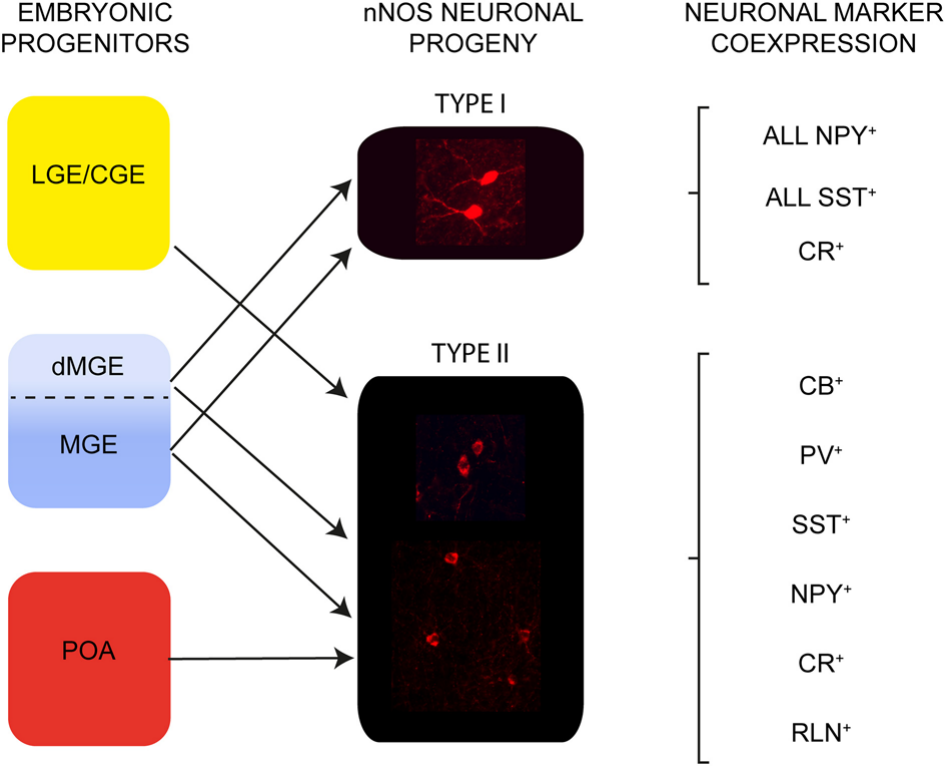
**The embryonic origin and neurochemical content of nNOS interneurons in the adult somatosensory mouse cortex.** Type I cells originate in the MGE. Type II cells have a triple origin in the LGE/CGE, MGE and POA. All type I cells coexpress SST and NPY and some coexpress CR. Heterogeneity of neurochemical marker expression is observed in type II cells.

We examined the development and origin of nNOS type I and type II interneurons in the neocortex. The two populations of nNOS neurons have different origins within the embryonic telencephalon: all type I cells are derived from the MGE whereas type II cells have a triple MGE, LGE/CGE, and POA origin (Figure 7). Neurogenesis of type I cells takes place during a narrower time-window compared to type II cells. Layer acquisition for both populations occurs in an inside-out manner and is dependent on birthplace. nNOS neurons are more abundant in the adult cortex than previously thought and represent ~20% of the entire cortical interneuron population. Type I and type II nNOS neurons have different distributions within the adult cortex. Most type I cells are located within lower cortical layers whereas type II cells are distributed in all layers. All type I cells in the mouse somatosensory cortex coexpress NPY and SST and about half express CR. Type I cells do not express CB, PV, or RLN (Figure 7). Type II cells show some coexpression with all markers examined (Figure 7). Our data confirm the previously described heterogeneity of the nNOS interneuron population based on the levels of nNOS expression, morphology, and coexpression of other interneuron markers. Our fate-mapping suggests that this heterogeneity is laid down during embryonic development when these cells are specified from neuroepithelial precursor cells.

### nNOS cortical interneurons are born early but upregulate nNOS just before birth

We detected putative nNOS cortical interneurons in the cortex only at E18.5 whereas most interneurons in the cortex are born earlier during embryogenesis (Fairen et al., 1986; Rymar and Sadikot, 2007). We demonstrate that both type I and type II cells are born early during gestation and suggest that they upregulate nNOS well after they enter the cortex. We cannot exclude the possibility of a subpallial delay in their migration and a late invasion of the cortex. However, an earlier marker that identifies these cells is required to address this question. Interestingly, we found that most type I cells were born at E12.5 whereas neurogenesis of type II cells spanned a wider time-window. This is in line with their embryonic origin: type I cells are all MGE-derived whereas type II cells have a triple origin in the MGE, LGE/CGE, and POA. The three germinal zones generate cortical interneurons at different times during development (Miyoshi et al., 2007, 2010; Gelman et al., 2009, 2011).

### Differential distribution and abundance of nNOS type I and type II interneurons in the adult cortex

In mice and monkeys the two types of nNOS neurons are distributed differently within cortical layers, with type I cells found mainly in deeper layers and the white matter and type II cells found throughout the cortex, especially layers II/III (Hashikawa et al., 1994; Yan et al., 1996; Smiley et al., 2000; Gotti et al., 2005; Lee and Jeon, 2005; Rockland and Nayyar, 2012). In rats however, the distribution seems to vary according to the area examined with type I cells being abundant in deep layers in the frontal cortex (Kubota et al., 1994) and in superficial layers in the visual cortex (Gonchar and Burkhalter, 1997). Our analysis was focused on the mouse somatosensory cortex and we found a consistent abundance of type I cells in lower layers (highest in layer VI) and in the white matter and type II cells in upper (II/III) and lower layers (VI). As described in other species, type II cells were always more numerous than type I cells.

### nNOS type I cells coexpress NPY, SST, and CR but not CB, PV, or RLN whereas subpopulations of type II cells show expression of all markers examined

In rats, monkeys, and rabbits nearly all type I cells coexpress SST and NPY and a large number express substance P receptor, whereas hardly any express PV or CR (Dawson et al., 1991; Kubota et al., 1994, 2011; Gonchar and Burkhalter, 1997; Smiley et al., 2000; Lee and Jeon, 2005; Karagiannis et al., 2009). Expression of PV and CR in type I cells in the mouse is controversial (Lee and Jeon, 2005; Gonchar et al., 2007) and CB expression varies in different cortical areas and across species (Kubota et al., 1994; Gonchar and Burkhalter, 1997; Smiley et al., 2000; Lee and Jeon, 2005). We found that all nNOS type I cells in the mouse somatosensory cortex coexpress SST and NPY and ~60% coexpress CR. We did not find any type I cells co-localizing with CB, PV, or RLN. In contrast, small numbers of type II cells coexpressed CB, CR, PV, SST, RLN, and to a greater extent NPY. This heterogeneity of marker expression observed within the type II population is consistent with findings in rats where expression of NPY and PV has been observed albeit not SST (Karagiannis et al., 2009; Kubota et al., 2011). In monkeys some expression of SST and NPY in type II cells has been detected although not CB, CR, or PV (Smiley et al., 2000). These differences in co-localization studies for type I and type II cells may reflect differences amongst species, cortical areas, methodology used or technical differences in detecting and distinguishing type II from type I cells.

### Distinct origins for nNOS type I and type II interneurons in the embryonic telencephalon

The embryonic origin of cortical nNOS interneurons has not been established. Analysis of mice lacking the MGE transcription factor NKX2.1 showed a complete absence of nNOS-expressing cells at prenatal stages suggesting an MGE-origin (Anderson et al., 2001) and lineage tracing in the POA identified some nNOS cortical interneurons being derived from this region (Gelman et al., 2009, 2011). However, no distinction had been made between type I and type II cells in either of these two studies. We used a series of transgenic mouse lines that allowed us to fate map the three subpallial sources of interneurons and we demonstrate that type I and type II cells have different origins: type I nNOS interneurons are generated exclusively from the MGE whereas type II cells have multiple origins in the MGE, LGE/CGE, and the POA. This is in agreement with a recent fate-mapping study that showed an exclusive MGE origin of nNOS type I cells (Jaglin et al., 2012). Type II cells had not been examined (Jaglin et al., 2012).

Using our transgenic mice we were able to subdivide the MGE and directly fate-map the dMGE and more ventral MGE regions. We found that the dMGE which expresses high levels of *Nkx6.2*, generates a large number of nNOS type I cells. Our previous finding that all SST^+^CR^+^ interneurons for the cortex originate in the dMGE (Fogarty et al., 2007) suggests that type I cells generated within this region include the entire CR-expressing population. Type I cells that do not express CR may originate from more ventrally-located MGE precursors. We demonstrate that *Nkx2.1-Cre*-generated type I cells occupy lower layers whereas *Nkx6.2-Cre*-derived cells are more evenly distributed across the layers. Altogether, the data indicate that CR^+^ and CR^−^ type I cells have different origins within the MGE and their layer distribution within the cortex is dependent on their birthplace.

Whilst most type I cells were found to be settled in lower layers in the adult cortex, type II cells were found in upper and lower layers. Layer selection for type II cells was dependent on the origin because MGE-derived type II cells had a bias for the lower layers and LGE/CGE and POA-derived cells were more abundant in upper layers. This is in line with previous genetic fate-mapping studies that showed distinct distribution patterns of interneurons originating in the three subcortical germinal zones (Miyoshi et al., 2007, 2010; Gelman et al., 2009, 2011; Rubin et al., 2010).

### Functional heterogeneity of type I and type II cells in the neocortex

Very little is known about the function and participation of nNOS interneurons in cortical circuits. An axo-dendritic subcellular targeting has been described but no distinction has been made between type I and type II cells (Seress et al., 2005). The two populations in the rat somatosensory cortex have distinct physiological features (Karagiannis et al., 2009). Our findings that type I and type II cells have different embryonic origins and settle in different layers of the cortex are consistent with the notion that the two cohorts represent distinct interneuron subtypes.

Recent evidence has shown that nNOS type I neurons are projecting neurons (Tomioka et al., 2005; Higo et al., 2009; Tamamaki and Tomioka, 2010) and form a population of neurons that are activated during sleep (Gerashchenko et al., 2008; Kilduff et al., 2011). We find that type I cells share common features: they are all generated from MGE precursors during a narrow neurogenesis window and coexpress NPY and SST. However, even type I cells may represent functionally distinct cell types given that (a) they originate from the *Nkx6.2*-expressing region in the dMGE as well as more ventrally-located MGE neuroepithelial cells, (b) they occupy different layers according to their origin, and (c) they show heterogeneity in CR expression. Detailed analysis of CR^+^ and CR^−^ SST-expressing Martinotti cells had found significant differences in their morphology and intrinsic physiology (Xu et al., 2006). This suggests that nNOS^+^SST^+^CR^+^ and nNOS^+^ SST^+^CR^−^ type I cells may differ in their morphological and/or electrophysiological features. Their differential distribution across layers may also be suggestive of distinct roles in cortical networks.

Hippocampal Ivy cells and a subpopulation of nNOS-expressing neurogliaform cells are two examples of MGE-derived interneuron subpopulations that have similar molecular profiles and common intrinsic physiological and morphological characteristics. Yet, the two populations reside in different layers in the hippocampus and participate at different times during network function, suggesting that they may represent functionally distinct interneuron subtypes (Price et al., 2005; Fuentealba et al., 2008; Szabadics and Soltesz, 2009; Tricoire et al., 2010, 2011). Whether Ivy cells and neurogliaform cells of the hippocampus have different origins within the MGE or have diverged functionally because of their differential lamination is unknown.

Within the type II population there is diversity in terms of birthdate, embryonic origin, laminar distribution and neurochemical content indicating that type II cells represent a heterogenous pool of neurons. nNOS type II cells coexpressing RLN in the cortex may correspond to a subpopulation of late-spiking neurogliaform cells that originate in the CGE (Lee et al., 2010; Miyoshi et al., 2010). To date cortical and hippocampal interneurons that have similar properties have been found to originate within the same progenitor zone. Late-spiking neurogliaform cells are an exemption to this because cortical neurogliaform cells originate within the CGE whereas hippocampal neurogliaform cells have a dual MGE/CGE origin (Lee et al., 2010; Miyoshi et al., 2010; Tricoire et al., 2010, 2011). Alternatively, there might be an MGE-derived neurogliaform cell in the cortex that has yet to be identified.

Interneurons that originate from the three major subpallial sources clearly have distinct neurochemical, morphological, and electrophysiological profiles. These are likely to be genetically imposed at their birthplace. Dissection of genetic specification pathways that confer subtype identity will provide insight into the development and ultimately the function of these cells in cortical circuits.

### Conflict of interest statement

The authors declare that the research was conducted in the absence of any commercial or financial relationships that could be construed as a potential conflict of interest.
